# Medication use before and during pregnancy in Japan: the Tohoku Medical Megabank Project Birth and Three-Generation Cohort Study

**DOI:** 10.1007/s00228-024-03685-7

**Published:** 2024-04-17

**Authors:** Aoi Noda, Taku Obara, Matsuyuki Shirota, Fumihiko Ueno, Fumiko Matsuzaki, Rieko Hatanaka, Ryo Obara, Kei Morishita, Genki Shinoda, Masatsugu Orui, Keiko Murakami, Mami Ishikuro, Shinichi Kuriyama

**Affiliations:** 1 Tohoku Medical Megabank Organization, Tohoku University, 2-1 Seiryou-Cho, Aoba-Ku, Sendai, Miyagi 980-8573 Japan; 2 Tohoku University Graduate School of Medicine, Sendai, Miyagi Japan; 3 Department of Pharmaceutical Sciences, Tohoku University Hospital, Sendai, Miyagi Japan; 4 International Research Institute of Disaster Science, Tohoku University, Sendai, Miyagi Japan

**Keywords:** Pregnancy, Medication use, Birth cohort, Teratogenic medication, Immunosuppressants, Antiepileptic medications

## Abstract

**Purpose:**

To elucidate the status of medication use among pregnant women in Japan, by means of a multigenerational genome and birth cohort study: the Tohoku Medical Megabank Project Birth and Three-Generation Cohort Study (TMM BirThree Cohort Study).

**Methods:**

Questionnaires were distributed to pregnant women participating in the TMM BirThree Cohort Study (from July 2013 to March 2017) around 12 weeks (early pregnancy) and 26 weeks (middle pregnancy). We analysed medication use over three periods: (1) 12 months prior to pregnancy diagnosis, (2) the period between pregnancy diagnosis and around week 12 of pregnancy, and (3) post around week 12 of pregnancy.

**Results:**

In total, 19,297 women were included in the analysis. The proportion of pregnant women using medications was 49.0% prior to pregnancy diagnosis, 52.1% from diagnosis to week 12, and 58.4% post week 12 of pregnancy. The most frequently prescribed medications were loxoprofen sodium hydrate (5.5%) prior to pregnancy diagnosis, magnesium oxide (5.9%) from diagnosis to week 12, and ritodrine hydrochloride (10.5%) post week 12 of pregnancy. The number of women who used suspected teratogenic medications during early pregnancy was 96 prior to pregnancy diagnosis, 48 from diagnosis to week 12, and 54 post week 12 of pregnancy.

**Conclusion:**

We found that ~ 50% of the pregnant women used medications before and during pregnancy and some took potential teratogenic medications during pregnancy. In birth genomic cohort study, it is expected that investigations into the safety and effectiveness of medications used during pregnancy will advance.

## Introduction

Information regarding the safety and effectiveness of medications in pregnant women is limited. Medications undergo extensive research, including preclinical testing and clinical trials, to ensure their safety and effectiveness in target populations [1]. However, such studies are rarely performed on pregnant women, to protect the foetus from research-related risks [2]. Results on medication safety and efficacy are obtained from animal studies, case reports, and observational studies. It is therefore difficult to confirm a causal relationship between foetal exposure to medication and adverse effects [3]. The rate of major congenital malformations in the general population ranges from 1.6 to 3.2% [4]. However, whether medication exposure during pregnancy causes these congenital anomalies is difficult to ascertain.

Previous studies have shown that 40–90% of women in developed countries take at least one medication during pregnancy [5–11]. When administering medication, it is important to pay careful attention to the side effects experienced by pregnant women, as well as those experienced by the foetus. However, excessive concerns about the risk of using medication may discourage pregnant women from adhering to beneficial treatments or refraining from necessary medication treatments, which may worsen maternal and infant health conditions [12, 13]. Particularly, the continuation of treatments with suspected teratogenic effects such as antiepileptic medications and immunosuppressants in pregnant patients is regarded as an issue. However, the need for continued medical treatment has been highlighted because there are many consultations for pregnant women and women who wish to become pregnant regarding the continued medical treatment [14]. The risks and benefits of providing appropriate medical care for pregnant women must be considered. In several European countries, such as Denmark and Sweden, a Medical Birth Registry, which includes prescriptions, hospitalisation, and birth information, is used to collect information on medication exposure during pregnancy and evaluate its safety and effects [15–21]. However, there is no such database in Japan, making it difficult to assess the safety and effectiveness of medications used during pregnancy.

The safety and effectiveness of each medication differ and depend on the specific metabolic enzymes present. To appropriately examine the safety and effectiveness of medication during pregnancy, it is necessary to collect detailed information on the medication used and the patient’s genomic data. For the purpose of obtaining basic data for evaluating the safety of medication use during pregnancy, this study aimed to elucidate the status of medication use among pregnant women who participated in the Birth and Three-Generation Cohort Study, a multigenerational genome and birth cohort study, conducted in Japan.

## Methods

### Study setting and participants

This study was based on the data obtained from the Tohoku Medical Megabank Project Birth and Three-Generation Cohort Study (TMM BirThree Cohort Study). The TMM BirThree Cohort Study is a prospective cohort study based in Miyagi (Japan) which collects information from questionnaires and biological samples, including genetic information. Furthermore, the information on both mothers and infants was linked. This study was published elsewhere [22–24]. Pregnant women and their family members were contacted at obstetric clinics or hospitals between 2013 and 2017; 23,406 pregnant women participated in the study. Written informed consent was obtained from all the participants. All participants were free to decline to participate in the study and were advised that there was no disadvantage or risk involved in their refusal to participate. The eligibility criteria for participants (expectant mothers) were as follows: (1) they should reside in the study area at the time of recruitment and (2) they should be able to comprehend Japanese and complete the self-administered questionnaire. The TMM BirThree Cohort Study protocol was approved by Tohoku University and the Tohoku Medical Megabank Organization Internal Review Board (2013–1-103–1).

### Data collection

All data on medications used during the TMM BirThree Cohort Study were obtained from two questionnaires: the first questionnaire was received by pregnant women around 12 weeks (early pregnancy), and the second was received around 26 weeks (middle pregnancy). Most questionnaires were distributed and collected by trained genome medical research coordinators when conducting face-to-face interviews with pregnant women participating in the recruitment process at approximately 50 obstetric clinics and hospitals in Miyagi. To evaluate drug use during organogenesis, we analysed data from the following three periods: (1) 12 months prior to pregnancy diagnosis (prior to pregnancy diagnosis), (2) the period between pregnancy diagnosis and around week 12 of pregnancy (from diagnosis to week 12), and (3) post around week 12 of pregnancy (post week 12 of pregnancy). During the periods (1) and (2), data on medication usage was collected using questionnaires in early pregnancy. We analysed the questionnaires completed by pregnant women. Medication names reported by the participants were frequently ambiguous, making the true medications used often difficult to ascertain. Multiple pharmacists and a medical doctor assessed the self-reported medication names and compared them to generic names using the Kyoto Encyclopaedia of Genes and Genomes MEDICUS [25]. Furthermore, multiple pharmacists examined the method of obtaining medications and reclassified them into prescribed medications and others. Medication use information over each period was collected. The suspected teratogenic medications used, specifically antiepileptic medications and immunosuppressants, were aggregated over each period. Teratogenic medications were defined using the 2017 Japanese guidelines for obstetric practice as follows: in early pregnancy, etretinate, carbamazepine, thalidomide, cyclophosphamide, danazol, thiamazole, trimethadione, valproate, vitamin A (retinol), phenytoin, phenobarbital, mycophenolate, misoprostol, methotrexate, and warfarin; in second trimester of pregnancy, streptomycin, kanamycin, tetracycline antibiotic, angiotensin-converting enzyme inhibitor, angiotensin II receptor blocker, and misoprostol [26]. For antiepileptic medications, the daily dose was examined because of the dose-dependent risk of teratogenicity [27, 28]. Valproic acid was classified based on 600 mg/day (the recommended daily dose by Japanese guidelines) [29], and carbamazepine was classified based on 1000 mg/day (previously determined to have no teratogenic effects) [30]. Immunosuppressants, including corticosteroids, were also investigated as there have been reports on the teratogenicity of their use during pregnancy [31–33].

### Data analysis

Based on the three periods analysed, the frequency and proportion of pregnant women were calculated, and the following variables were considered: education, family income, smoking, alcohol consumption, childbirth delivery history, infertility treatment, history of epilepsy, and the presence of connective tissue diseases. We analysed the proportion of pregnant women who took one or more medications, over the counter (OTC) medications, or supplements. Statistical analyses were performed using the Statistical Analysis System (SAS) version 9.4 (SAS Institute Inc., Cary, NC, USA).

## Results

### Characteristics of study participants

**Fig. 1:**
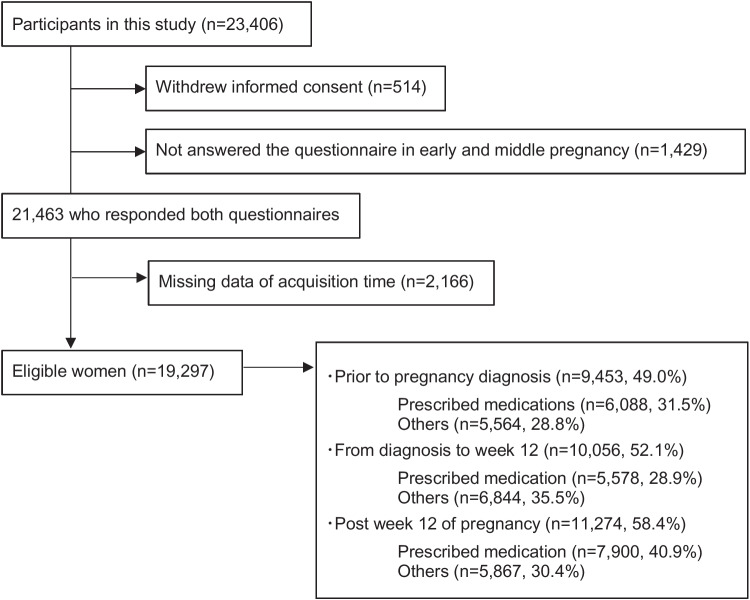
Flow chart displaying the exclusion criteria used for this study and the number of pregnant women using prescribed over-the-counter medications or supplements

 Data on 23,730 pregnant women, including those with multiple pregnancies, were collated from the TMM BirThree Cohort Study. Women were excluded from the study if they had refrained from answering both questionnaires, withdrew informed consent, or did not include information describing the time of medication acquisition. Consequently, 19,297 mothers were included in the final analysis (Fig. 1). Of them, 14,971 (77.6%) mothers reported using prescribed OTC medications or supplements during pregnancy. The mean age of pregnant women at the time of delivery was 31.4 ± 5.0 years. Furthermore, the proportion of pregnant women who used some form of medication during pregnancy was higher in more educated, higher income communities, and in those who had infertility treatment (Table 1). The proportion of pregnant women who used some form of medication prior to pregnancy diagnosis was higher in those who had infertility treatment. The proportion of pregnant women who used medications was 49.0% prior to pregnancy diagnosis, 52.1% from diagnosis to week 12, and 58.4% post week 12 of pregnancy (Fig. 1).

**Table 1:** Characteristics of the study population

	Total (*n*=19,297)		Prior to pregnancy diagnosis^a^ (*n*=9,453)		From diagnosis to week 12^a^ (*n*=10,056)		Post week 12 of pregnancy^a^ (*n*=11,274)		*P*
	*n*	%	*n*	%	*n*	%	*n*	%	
Mother’s age, mean (SD), y	31.4±5.0		32.1±4.8		32.1±4.8		32.2±4.8		0.9919
Educational attainment									
High school graduate or less	4,108	21.3	1,842	19.5	1,998	19.9	2,262	20.1	0.1881
Junior or vocational college graduate	4,801	24.9	2,598	27.5	2,690	26.8	3,026	26.8	
University graduate or above	3,589	18.6	2,188	23.2	2,284	22.7	2,468	21.9	
Missing	6,799	35.2	2,825	29.9	3,084	30.6	3,518	31.2	
Household income (JPY/year)									
< 4,000,000	6,716	34.8	2,889	30.6	3,168	31.5	3,562	31.6	0.5447
4,000,000 to < 6,000,000	5,930	30.7	2,980	31.5	3,195	31.8	3,534	31.4	
≥ 6,000,000	5,651	29.3	3,176	33.6	3,254	32.4	3,680	32.6	
Missing	1,000	5.2	408	4.3	439	4.4	498	4.4	
Infertility treatment									
Yes	1,926	10.0	1,397	14.8	1,325	13.2	1,412	12.5	<0.0001
No	17,310	89.7	8,035	85.0	8,707	86.6	9,828	87.2	
Missing	61	0.3	21	0.2	24	0.2	34	0.3	
Cigarette smoking									
Never	11,540	59.8	5,953	63.0	6,313	62.8	6,982	61.9	0.258
Stopped before pregnancy	4,472	23.2	2,257	23.9	2,382	23.7	2,660	23.6	
Stopped after pregnancy	2,733	14.2	1,064	11.3	1,165	11.6	1,390	12.3	
Smoking at early pregnancy	468	2.4	150	1.6	173	1.7	206	1.8	
Missing	84	0.4	21	0.2	24	0.2	34	0.3	
Alcohol consumption									
Drinking at early pregnancy	3,709	19.2	1,952	20.7	1,891	18.8	685	6.1	<0.0001
Former	6,614	34.3	3,350	35.4	3,747	37.3	4,689	41.6	
Never	7,783	40.3	3,588	38.0	3,808	37.9	5,131	45.5	
Cannot drink because of constitution	1,124	5.8	542	5.7	582	5.8	742	6.6	
Missing	67	0.4	21	0.2	28	0.3	27	0.2	
Delivery history	10,300	53.4	4,401	46.6	4,911	48.8	5,582	49.5	<0.0001
Epilepsy	94	0.5	62	0.7	70	0.7	74	0.7	0.9219
Connective tissue disease	97	0.5	74	0.8	77	0.8	81	0.7	0.8548
Collagen disease	23	0.1	15	0.2	16	0.2	21	0.2	0.8533
Autoimmune disorder	20	0.1	16	0.2	18	0.2	14	0.1	0.5539
Systemic lupus erythematosus	20	0.1	16	0.2	17	0.2	18	0.2	0.9807
Rheumatoid arthritis	34	0.2	27	0.3	26	0.3	28	0.2	0.8676
The use of supplements or OTC	9,220	47.8	5,564	58.9	6,844	68.1	5,867	52.0	<0.0001

*SD* Standard deviation, *OTC* Over-the-counter

^a^We analysed data from the following three periods: (1) 12 months prior to pregnancy diagnosis (prior to pregnancy diagnosis), (2) the period between pregnancy diagnosis and around week 12 of pregnancy (from diagnosis to week 12), and (3) post around week 12 of pregnancy (post week 12 of pregnancy)

### Medications used during prenatal period

The most frequently prescribed medications were loxoprofen sodium hydrate (5.5%) used prior to pregnancy diagnosis, magnesium oxide (5.9%) from diagnosis to week 12, and ritodrine hydrochloride (10.5%) post week 12 (Table 2).

**Table 2:** Medication use before and during pregnancy (*n* = 19,297)

Prior to pregnancy diagnosis^a^			From diagnosis to week 12^a^			Post week 12 of pregnancy^a^		
General name	*n*	%	General name	*n*	%	General name	*n*	%
Loxoprofen sodium hydrate	1064	5.5	Magnesium oxide	1,138	5.9	Ritodrine hydrochloride	2024	10.5
l-Carbocisteine	815	4.2	Acetaminophen	747	3.9	Magnesium oxide	1910	9.9
Acetaminophen	663	3.4	Piperidolate hydrochloride	544	2.8	Sodium ferrous citrate	1054	5.5
Tranexamic acid	561	2.9	Ritodrine hydrochloride	471	2.4	Acetaminophen	663	3.4
Clarithromycin	481	2.5	Tranexamic acid	361	1.9	Sodium picosulfate hydrate	404	2.1
Fexofenadine hydrochloride	422	2.2	Sodium ferrous citrate	310	1.6	Shoseiryuto extract	294	1.5
Salicylamide, acetaminophen, anhydrous caffeine, and promethazine methylenedisalicylate	350	1.8	l-Carbocisteine	272	1.4	l-Carbocisteine	286	1.5
Cefcapene pivoxil hydrochloride hydrate	336	1.7	Sodium picosulfate hydrate	271	1.4	Sennosides	269	1.4
Clomifene citrate	324	1.7	Carbazochrome sodium sulfonate hydrate	270	1.4	Heparinoid	209	1.1
Rebamipide	323	1.7	Shoseiryuto extract	262	1.4	Hydrocortisone and killed *Escherichia coli* suspension	176	0.9
Magnesium oxide	311	1.6	Heparinoid	213	1.1	Hydrocortisone and killed *Escherichia coli* suspension	176	0.9
Olopatadine hydrochloride	288	1.5	Kakkonto extract	213	1.1	Salicylamide, acetaminophen, anhydrous caffeine, and promethazine methylenedisalicylate	174	0.9
Heparinoid	255	1.3	Isoxsuprine hydrochloride	197	1.0	Bifidobacterium	174	0.9
Levofloxacin hemihydrate	255	1.3	Salicylamide, acetaminophen, anhydrous caffeine, and promethazine methylenedisalicylate	166	0.9	Dimemorfan phosphate	165	0.9
Cefditoren pivoxil	249	1.3	Aspirin and dialuminate; aspirin, aluminium glycinate, and magnesium carbonate	163	0.8	Kakkonto extract	162	0.8

^a^We analysed data from the following three periods: (1) 12 months prior to pregnancy diagnosis (prior to pregnancy diagnosis), (2) the period between pregnancy diagnosis and around week 12 of pregnancy (from diagnosis to week 12), and (3) post around week 12 of pregnancy (post week 12 of pregnancy)

The number of pregnant women who used suspected teratogenic medications in early pregnancy was 96 (50.0/10,000 deliveries) prior to pregnancy diagnosis, 48 (25.2/10,000 deliveries) from diagnosis to week 12, and 54 (28.0/10,000 deliveries) post week 12. The most frequently used teratogenic medications were antiepileptic medications such as valproate, carbamazepine, and phenytoin. The number of pregnant women using the aforementioned teratogenic medications during the second trimester was 105 (54.4 per 10,000 deliveries); however, most pregnant women did not use them after receiving a diagnosis of pregnancy (Table 3). Table 4 shows the maximum doses of valproate and carbamazepine received before and during pregnancy. One (5.9%) of the 17 women used valproate at < 600 mg/day from diagnosis to week 12. Carbamazepine (< 1000 mg/day) was administered to ten women from diagnosis to week 12. The maximum dose of carbamazepine administered was 800 mg/day. The proportion of medications used decreased after a pregnancy diagnosis (Table 4).

**Table 3:** The number of pregnant women using suspected teratogenic medications* before and during pregnancy (*n* = 19,297)

General name	Prior to pregnancy diagnosis^a^	From diagnosis to week 12^a^	Post week 12 of pregnancy^a^
	*n* (per 10,000)	*n* (per 10,000)	*n* (per 10,000)
Medications suspected to be teratogenic when used in early pregnancy			
Total	96 (50.0)	48 (25.2)	54 (28.0)
Valproate	39 (20.2)	17 (8.8)	20 (10.4)
Thiamazole	27 (14.0)	10 (5.2)	15 (7.8)
Carbamazepine	21 (10.9)	14 (7.3)	13 (6.7)
Phenytoin	4 (2.1)	4 (2.1)	4 (2.1)
Methotrexate	2 (1.0)	0 (0.0)	0 (0.0)
Phenobarbital	2 (1.0)	2 (1.0)	1 (0.5)
Phenytoin and phenobarbital	1 (0.5)	1 (0.5)	1 (0.5)
Medications suspected to be teratogenic when used in second trimester			
Total	105 (54.4)	6 (3.1)	2 (1.0)
Doxycycline hydrochloride hydrate	56 (29)	1 (0.5)	0 (0.0)
Minocycline hydrochloride	35 (18.1)	4 (2.1)	1 (0.5)
Candesartan cilexetil	5 (2.6)	1 (0.5)	0 (0.0)
Valsartan	3 (1.6)	0 (0.0)	0 (0.0)
Tetracycline hydrochloride	2 (1)	0 (0.0)	1 (0.5)
Azilsartan	1 (0.5)	0 (0.0)	0 (0.0)
Irbesartan	1 (0.5)	0 (0.0)	0 (0.0)
Telmisartan	1 (0.5)	0 (0.0)	0 (0.0)
Losartan potassium	1 (0.5)	0 (0.0)	0 (0.0)

*In guidelines for obstetrical practice in Japan 2017 edition, the suspected teratogenic medications are listed as follows: in early pregnancy, etretinate, carbamazepine, thalidomide, cyclophosphamide, danazol, thiamazole, trimethadione, valproate, vitamin A (retinol), phenytoin, phenobarbital, mycophenolate, misoprostol, methotrexate, and warfarin; in second trimester, streptomycin, kanamycin, tetracycline antibiotic, angiotensin-converting enzyme inhibitor, angiotensin II receptor blocker, and misoprostol

^a^We analysed data from the following three periods: (1) 12 months prior to pregnancy diagnosis (prior to pregnancy diagnosis), (2) the period between pregnancy diagnosis and around week 12 of pregnancy (from diagnosis to week 12), and (3) post around week 12 of pregnancy (post week 12 of pregnancy)

**Table 4:** The daily dose of valproate and carbamazepine

mg/day	Prior to pregnancy diagnosis^a^	From diagnosis to week 12^a^	Post week 12 of pregnancy^a^
Valproate			
600 <	27	13	14
600–1000	4	1	3
> 1000	0	0	0
Missing	8	3	3
Carbamazepine			
1000 ≤	17	10	11
> 1000	0	0	0
Missing	4	4	2

^a^We analysed data from the following three periods: (1) 12 months prior to pregnancy diagnosis (prior to pregnancy diagnosis), (2) the period between pregnancy diagnosis and around week 12 of pregnancy (from diagnosis to week 12), and (3) post around week 12 of pregnancy (post week 12 of pregnancy)

The proportion of immunosuppressants and corticosteroids used before and during pregnancy are shown in Table 5. Tacrolimus hydrate was the most frequently used immunosuppressant. None of the patients used immunosuppressants, other than tacrolimus, after a pregnancy diagnosis.

**Table 5:** The status of corticosteroid and immunosuppressant use before and during pregnancy

Generation name	Prior to pregnancy diagnosis^a^	From diagnosis to week 12^a^	Post week 12 of pregnancy^a^
	*n* (per 10,000)	*n* (per 10,000)	*n* (per 10,000)
Corticosteroid	184 (95.4)	118 (61.1)	98 (50.8)
Prednisolone (oral)	106 (57.5)	81 (44.6)	62 (39.4)
Prednisolone (topical)	5 (2.6)	6 (3.1)	14 (7.3)
Dexamethasone (oral)	16 (8.3)	3 (1.6)	0 (0.0)
Dexamethasone (topical)	25 (13.0)	17 (8.8)	10 (5.2)
Triamcinolone acetonide (topical)	23 (11.9)	9 (4.7)	11 (5.7)
Betamethasone	5 (2.6)	1 (0.5)	0 (0.0)
Hydrocortisone	1 (0.5)	1 (0.5)	1 (0.5)
Hydrocortisone sodium succinate	1 (0.5)	1 (0.5)	0 (0.0)
Methylprednisolone	1 (0.5)	0 (0.0)	0 (0.0)
Methylprednisolone sodium succinate	1 (0.5)	0 (0.0)	0 (0.0)
Immunosuppressant	44 (22.8)	11 (5.7)	7 (3.6)
Tacrolimus hydrate	38 (19.7)	11 (5.7)	7 (3.6)
Methotrexate	2 (1.0)	0 (0.0)	0 (0.0)
Ciclosporin	2 (1.0)	0 (0.0)	0 (0.0)
Mizoribine	2 (1.0)	0 (0.0)	0 (0.0)

^a^We analysed data from the following three periods: (1) 12 months prior to pregnancy diagnosis (prior to pregnancy diagnosis), (2) the period between pregnancy diagnosis and around week 12 of pregnancy (from diagnosis to week 12), and (3) post around week 12 of pregnancy (post week 12 of pregnancy)

## Discussion

The proportion of pregnant women who used at least one medication, supplement, or OTC medication from diagnosis to week 12 and post week 12 of pregnancy was 49.0% and 52.1%, respectively. These percentages were lower than those reported in similar studies carried out in Germany (69.7%, 80.7%) [8], France (76.4%, 81.1%) [5], and Italy (59.1%, 53.1%) [6] and higher than those reported in Norway (35.0%, 30.6%) [10]. However, it is difficult to draw direct comparisons between these studies because of the methodological differences, difficulties in ascertaining the medicines used, and variations in the medication categories [34]. When using a prescription database, it is important to take into consideration that these databases may overestimate the actual medication usage, since not all prescribed medications will be consumed by the patient. In contrast, retrospective studies are likely to underreport past events such as medication usage [34, 35]. Bearing these limitations in mind, in our study, the proportion of pregnant women who used at least one medication, supplement, or OTC medication in early to middle pregnancy was found to be moderate when compared to previous studies conducted in other countries. We noted that medication usage increased in older women with high educational and socioeconomic status, and this trend was similar to those observed in previous studies [12, 34, 36].

Before the diagnosis of pregnancy, medications related to the common cold were frequently used; however, after a pregnancy diagnosis, various medications such as laxatives, iron preparations, traditional Japanese (Kampo) medicine, and ritodrine were used. Kampo medicine, such as shoseiryuto and kakkonto, may be used instead of antibiotics to treat colds during pregnancy [7, 37]. They originate from traditional Chinese medicine, and it is widely used together with modern medicine in clinical settings, including in obstetrics [37–40]. Kampo medicines have been shown to be more acceptable to pregnant women because of the perception that they are natural and do not pose adverse effects, unlike conventional medicines [41–43]. The use of tranexamic acid and carbazochrome sodium sulfonate hydrate from diagnosis to week 12 might be conventionally used for the treatment of threatened miscarriage. The proportion of pregnant women who had used ritodrine hydrochloride, a uterine relaxant, from diagnosis to week 12 was 2.4%, which increased to 10.5% post week 12 of pregnancy. In 2013, the European Medicines Agency restricted the use of ritodrine in Europe because of its associated cardiovascular risks and little data to support the short- or long-term benefits of its use as a uterine relaxant; oral ritodrine is no longer available for pregnant women [44]. However, in Japan, ritodrine is still used during the early stages of pregnancy and is used more frequently than in other countries. Therefore, it is necessary to clarify the efficacy and safety of ritodrine in pregnant women in Japan.

The proportion of women using suspected teratogenic medications decreased after a pregnancy diagnosis. However, some of these medications were used even after pregnancy was diagnosed. In this study, the prevalence of valproate use decreased to less than 50% from diagnosis to week 12, a trend similar to that reported in a previous study conducted in Japan [7]. Some women who used valproate prior to pregnancy diagnosis did not use it during pregnancy. One pregnant woman who used 800 mg/day of valproate prior to pregnancy diagnosis switched to lamotrigine at an unknown dosage after pregnancy diagnosis. Two pregnant women who had used valproate at an unknown dosage prior to pregnancy diagnosis stopped using valproate after a pregnancy diagnosis and restarted 400 and 800 mg/day of valproate, respectively, post week 12 of pregnancy. This might suggest that valproate treatment could be suspended or replaced, as recommended by regulatory agencies [29, 45–47]. However, the use of these medications is unavoidable in pregnant women. In several studies, it has been shown that valproate has a dose-dependent risk associated with major congenital malformations and neurodevelopmental problems [27, 28]. The Japanese guidelines issued in 2018 recommend limiting the daily dose of valproate to 600 mg/day [29]. In this study, one pregnant woman continued to take 800 mg/day after the pregnancy diagnosis. However, most pregnant women used 600–1000 mg/day before the pregnancy diagnosis and replaced or suspended valproate after the diagnosis. Valproate may be administered at adjusted doses to avoid its use during early pregnancy.

The incidence of rheumatic diseases, particularly systemic lupus erythematosus (SLE), is the highest among women of reproductive age [48], and some pregnant women may use corticosteroids or immunosuppressants. Previous studies have shown that corticosteroid use during early pregnancy increases the risk of oral cleft [31–33]. In this study, after pregnancy diagnosis, the number of women who took betamethasone or dexamethasone orally was reduced to one-fifth of that used pre-pregnancy. Betamethasone and dexamethasone have high placental passage [49, 50]. In Japan, the medication package inserts for three immunosuppressants (cyclosporine, tacrolimus hydrate, and azathioprine) were revised in 2018, and pregnant women were excluded from the contraindication section. In a retrospective study of pregnant women with SLE, immunosuppressant prescriptions during pregnancy decreased when compared to those prescribed prior to pregnancy [7, 44]. None of the women in this study used immunosuppressants, other than tacrolimus, after a pregnancy diagnosis. However, we suspect that due to the revised immunosuppressant contraindications, more women may continue immunosuppressants during pregnancy; physicians may prescribe immunosuppressants to pregnant women by closely monitoring the condition of the patient’s disease and weighing the expected therapeutic benefits against the possible risks associated with treatment. The revised package inserts are not intended to unconditionally allow the use of immunosuppressants in pregnant women [14]; it is therefore important to monitor the use of these medications in pregnant women in Japan.

This study included various limitations. First, the participants were limited to pregnant women who voluntarily participated in The TMM BirThree Cohort Study. Therefore, cooperative and health-conscious pregnant women were more likely to participate in this study, and this may have resulted in an underestimation of prescribed medications. Second, this study did not assess the validity of self-reported medication use. Previous studies in Japan showed that self-reported questionnaires on medication use have high validity [51–53]. These studies were not conducted in pregnant women; however, our study may have a higher validity because pregnant women are generally more concerned about the use of medication than the general population. Third, some data regarding the detailed daily doses of valproate and carbamazepine were missing. Pregnant women should be informed of the dose-dependent teratogenic risks associated with valproate and carbamazepine [27, 28]. By combining objective data such as claims databases with self-reported data or data collected from genome cohort study, it may be possible to compensate for missing data and appropriately evaluate teratogenic risk considering genomic data [54, 55]. Fourth, we could not collect data post around 26 weeks of pregnancy. This may limit the comparability with other studies. Fifth, it was not always possible to determine the dosage form from the medication names reported by the participants. Further research is needed to know the purpose of use.

## Conclusion

We found that ~ 50% of pregnant women in this study used medications before and during pregnancy, and some took teratogenic medications during pregnancy. It is critical to consider both the risks and benefits of providing appropriate medical care to pregnant patients. We can more precisely evaluate the safety of medication during pregnancy by considering and combining family, genomic, and environmental information. Future studies should investigate the factors behind the teratogenic effects on the foetus by linking the medication and genetic information of pregnant women and infants. 

## Data Availability

The data obtained through the TMM BirThree Cohort Study are incorporated into the TMM biobank. All data analysed during the present study are available for research purposes with the approval of the Sample and Data Access Committee of the Biobank.
